# 517. The Local Impact of Burn Injuries on the Bone – An Intraindividually Comparative Pilot Study

**DOI:** 10.1093/jbcr/irag033.176

**Published:** 2026-04-06

**Authors:** Maximilian Moshammer, Nikolaus Watzinger, Andrzej Hecker, Christian Smolle, Manuel Prevedel, Petra Kotzbeck, Lars-Peter Kamolz, Ines Foessl

**Affiliations:** COREMED – Center for Regenerative and Precision Medicine, JOANNEUM RESEARCH Forschungsgesellschaft mbH, Graz, Austria, Graz, Steiermark; Division of Plastic, Aesthetic and Reconstructive Surgery, Department of Surgery, Medical University of Graz, Austria, Graz, Steiermark; Medical University of Graz, Department of Surgery, Division of Plastic, Aesthetic and Reconstructive Surgery, Graz, Steiermark; Division of Plastic, Aesthetic and Reconstructive Surgery, Department of Surgery, Medical University of Graz, Austria, Graz, Steiermark; COREMED – Center for Regenerative and Precision Medicine, JOANNEUM RESEARCH Forschungsgesellschaft mbH, Graz, Austria, Graz, Steiermark; COREMED – Center for Regenerative and Precision Medicine, JOANNEUM RESEARCH Forschungsgesellschaft mbH, Graz, Austria, Graz, Steiermark; Division of Plastic, Aesthetic and Reconstructive Surgery, Department of Surgery, Medical University of Graz, Austria, Graz, Steiermark; COREMED – Center for Regenerative and Precision Medicine, JOANNEUM RESEARCH Forschungsgesellschaft mbH, Graz, Austria, Graz, Steiermark

## Abstract

**Introduction:**

Burn injuries trigger both local and systemic inflammation. While systemic effects such as hypermetabolism and generalized bone loss are well documented, little is known about whether bone directly beneath the burn site is affected.

**Methods:**

We investigated the local impact of hand burns on underlying bone using high-resolution peripheral quantitative computed tomography (HR-pQCT). Intraindividual comparisons were performed between the deepest burned site and radius of the affected hand with the corresponding unburned regions of the contralateral hand.

**Results:**

Nine patients were included, and their burned hand was intraindividually compared with the contralateral hand. Total volumetric bone mineral density (vBMD) was significantly reduced at the site of the deepest burn compared to the control hand (*p*=.035), with a strong effect size of -0.807 (95% CI: [-1.522, -0.056]). No difference was observed at the radius (*p*=.764). Additionally, medium effects of burn injuries were found for inner trabecular vBMD (60% of Tb.Ar; 95% CI: [-1.209, 0.143]), trabecular area (Tb.Ar; 95% CI: [-0.066, 1.324]), and cortical vBMD (95% CI: [-1.315, 0.072]). Mean grip strength of the burned hands was slightly higher at 42.6 kg (SD: 10.48) compared to 40.7 kg (SD: 9.73) of the control hands.

**Conclusions:**

This study provides the first clinical evidence of localized bone loss directly beneath burn sites, that extends beyond the systemic effects. Despite preserved grip strength, these findings highlight the need for early assessment and targeted interventions to preserve skeletal integrity following burns.

**Applicability of Research to Practice:**

Demonstrating localized bone loss in the hand after a burn highlights the need for early assessment and targeted interventions to preserve skeletal integrity in burn patients. This finding can inform clinical practice by guiding rehabilitation strategies and monitoring protocols to prevent long-term functional impairment.

**Funding for the study:**

N/A.

